# 1005 The Impact of a Therapy Dog on a Burn Provider’s Mood and Burnout

**DOI:** 10.1093/jbcr/iraf019.536

**Published:** 2025-04-01

**Authors:** Kristin Rainey, Kristin Rainey, Emily Snyder, Jennifer Rosenthal

**Affiliations:** Medical City Plano; Medical City Plano; Medical City Plano; Medical City Plano

## Abstract

**Introduction:**

Burnout is one of many contributing factors that lead to nurses and other medical staff leaving the bedside resulting in high turnover rates. In 2017, 31.5% of nurses who left bedside, left due to burnout. Additionally, over half of healthcare providers report burnout, which can impact stress, attendance, mood, productivity, and work quality.

Animal-assisted support programs can assist in the reduction of burnout in providers. In previous studies, all patients and staff reported improved mood after interacting with a therapy dog. Currently there is limited research in the effect of animal-assisted support programs in the hospital setting specifically regarding burn providers and burnout.

**Methods:**

A voluntary study was sent out to the staff members on the Burn Trauma Intensive Care Unit and the Ortho/Trauma Unit. These units were selected due to the large number of burn patients who are admitted in these areas. The study collected basic demographic data and asked the providers to rate their mood at that time using a visual analog scale. Additionally, the providers were asked to complete the Copenhagen Burnout Inventory (CBI). The CBI scores burnout in three categories: personal burnout, work-related burnout, and client-related burnout. The survey was available for two weeks and.

Following the survey, the therapy dog and handler completed 30-minute weekly visits to each unit. The visits occurred during day shift and the participants were asked to rate their mood prior to and after interacting with the therapy dog. Weekly visits continued for three months.

**Results:**

The initial survey received 42 responses. At the time of data collection, 34 interactions had been completed and the providers mood increased by an average of 76%. A paired t-test determined statistical significance with a p value < 0.001.

At the conclusion of the study, an additional survey will be sent out to the participants. This survey will collect the same data as the pre-survey along with the approximate number of interactions the staff member had with the therapy dog, and if they felt their mood would be increased if they knew a therapy dog would be available during their shift.

**Conclusions:**

Thus far the study has shown the staff’s mood improved immediately following the visit with the therapy dog. However, data is yet to be finalized to see if the therapy dog helps to decrease the burnout rates over time as indicated from the final CBI results.

**Applicability of Research to Practice:**

Scheduled visits with a therapy dog should be considered in burn units to improve staff mood and reduce caregiver burnout.

**Funding for the Study:**

N/A

